# Bovine ISG15: an antiviral and inducible protein in BIV infected fetal bovine lung cells

**DOI:** 10.1186/1743-422X-7-134

**Published:** 2010-06-23

**Authors:** Chang Liu, Xin Li, Xue Yao, Xiaohong Kong, Wentao Qiao, Yunqi Geng

**Affiliations:** 1 Key Laboratory of Molecular Microbiology and Biotechnology (Ministry of Education) and Key Laboratory of Microbial Function Genomics (Tianjin), College of Life Sciences, Nankai University; N0. 94, Rd. Weijin, Nankai District, Box. 300071, Tianjin, PR China; 2 School of Medicine, Nankai University, N0. 94, Rd. Weijin, Nankai District, Box. 300071, Tianjin, PR China

## Abstract

Bovine ISG15 (bISG15) is an interferon inducible ubiquitin-like protein that is responsible for the establishment of early pregnancy in ruminant, understanding the properties of bISG15 capable of being inducible in fetal bovine lung (FBL) cells upon infection of bovine immunodeficiency virus (BIV) is of significant importance. In this study, we investigated the expression of bISG15 in poly I:C treated FBL cells. The increased expression of bISG15 was observed, and the inhibition of BIV replication was also detected in FBL cells. Elimination of bISG15 expression by small interfering RNA reversed the bISG15 mediated inhibition of BIV replication. These findings demonstrate that bISG15 plays an important role in inhibition of the BIV replication in FBL cells. Furthermore, real-time PCR and western blot assay revealed that bISG15's expression can also be induced in BIV infected FBL cells. Taken together, bISG15 is an antiviral and inducible protein in BIV infected FBL cells.

## Finding

Type I IFNs' binding to cell type specific receptors can induce the synthesis of a plethora of proteins [1], many of which play crucial roles in the antiviral response [2]. ISG15 was one of the most strongly induced proteins. Thirty years past since ISG15 was first found [3], whereas its antiviral function was reported recently [4]. ISG15, a 15-kDa ubiquitin-like protein, can be covalently conjugated to target proteins for post-translational modification through a series of steps similar to ubiquitin modification [5]. ISG15 and its conjugation modification play roles in processes including regulation of IFN signaling, innate immunity, anti-viral infections, pregnancy, and carcinogenesis [4]. Interestingly, ISG15 and enzymes involved in ISG15 modification are interferon inducible [1,3,6-8]. This indicates ISG15 and its conjugation play important roles in innate immunity and antiviral response to infection. Mice deficient of ISG15 have been shown to be more susceptible to influenza, herpes, and Sindbis viruses' infection [9]. It has been reported that ISG15 can inhibit the release of human immunodeficiency virus type 1 (HIV-1) virions by disrupting the interaction of the Gag L domain with Tsg101 [10], and ISG15 can also repress the replication of Ebola virus by inhibiting the release of virions [11]. Unlike ubiquitin or other ubiquitin-like molecules, cross-species' conservation of ISG15 is low [4]. Therefore, it is necessary and intriguing to elucidate the antiviral functions of ISG15 in wide variety of species.

Bovine ISG15 (bISG15) has 70% homology amino acid sequence to human ISG15 [12]. The studies about bISG15 are mainly focused on its role in reproductive biology. The expression of bISG15 is up-regulated at endometrium in response to conceptus-secreted IFN-τ during early pregnancy [13]. The relation between bISG15 and innate immune response has also been noticed. The expression of bISG15 in maternal blood is increased in cows infected with acute non-cytopathic bovine viral diarrhea virus (BVDV) [14]. However, the reports about property of the bISG15 with antiviral function are scarce up to date. Recently, we have found that expression of bISG15 can be induced in fetal bovine lung (FBL) cells after treated with poly I:C or lipopolysaccaride (LPS) [15]. FBL cells were chosen to be as a target type of cells here, since our lab found that FBL cells could be infected by viruses which are capable of infecting cattle, such as BVDV, bovine foamy virus (BFV), and bovine immunodeficiency virus (BIV). BIV can infect FBL cells in vitro, and induce syncytia formation in FBL cells. Moreover, BIV DNA can also be detected in bovine lung tissues [16]. BIV, which has a similar genome to HIV-1, is associated with chronic inflammatory diseases in infected cows [17]. Considering the similarity between BIV and HIV-1, BIV may contribute as alternative animal virus model for certain aspects of HIV research. It has been reported that ISG15 can inhibit the replication of HIV [10]. We previously demonstrated that bISG15 can be induced robustly in poly I:C treated FBL cells [15]. Here, we further try to address whether the replication of BIV can be repressed in FBL cells treated with poly I:C and to address extensively whether the expression of bISG15 is changed in FBL cells infected with BIV.

In this study, FBL cells were isolated from fetal bovine lung issues. The primary FBL cells isolated from the lung issues of fetal calf were called passage"1". When FBL cells proliferated and separated, they were called passage "2" and so on. All the FBL cells used in the experiment were the passages between 10 and 20. BIV indicator cell line, designated as BIVL, has an integrated reporter plasmid which has a firefly luciferase reporter protein gene downstream of BIV LTR promoter [18]. FBL cells and BIVL cells were maintained in Dulbecco's Modified Eagle Medium (DMEM) supplemented with 10% fetal bovine serum (FBS), 50 IU/ml penicillin, 50 μg/ml streptomycin in 5% CO2 at 37°C. Due to fail of obtaining cell free BIV, a BIV strain R29 was used here. The BIV R29 strain was maintained in FBL cells. All the BIV used in the experiment were the FBL cells infected BIV. Poly I:C (purchased from Sigma-Aldrich) was dissolved in PBS at a concentration of 2.5 mg/ml. BIVL cells were plated at a density of 4 × 104 per well in 12-well plates to indicate BIV infection efficiency. BIVL cells co-cultured with BIV infected/uninfected FBL cells at equal cell number. After 12 hours, all cells were assessed by luciferase assays (Promega luciferase assay kit) to indicate the replication efficiency of BIV in FBL cells [18]. Synthetic siRNA of bISG15 (bISG15siRNA) and negative control siRNA (scrambled siRNA) (purchased from GenePharma) were used as annealed oligonucleotide and was resuspended in RNase-free H2O, respectively. The sense sequence of scrambled siRNA is 5'-UUC UCC GAA CGU GUC ACG UTT-3', the anti-sense sequence of scrambled siRNA is 5'-ACG UGA CAC GUU CGG AGA ATT-3'; and the sense sequence of bISG15siRNA is 5'-CAC CGU GUU CAU GAA UCU ATT-3', the anit-sense sequence of bISG15siRNA is 5'-UAG AUU CAU GAA CAC GGU GTT-3'. To validate the suppressing effect of bISG15, bISG15siRNA or scrambled siRNA (100 Nmol/hole) was transfected to FBL cells with Lipofectamine 2000 (Invitrogen), respectively. Realtime PCR and Western blot assay of bISG15 were performed as previously described [15]. Briefly, total RNA from cells was isolated with TRIzol reagent (Invitrogen) following the manufactures's instructions, and then the extracted RNA was reverse transcribed into cDNA by reverse transcriptase (Promega). Real time PCR was performed using an IQ5 Multicolor Real-time PCR detection system (Bio-Rad) and fluorescent EVAGreen nucleic acid stain (Biotium). Primers used to quantify bISG15's cDNA were bISG15-F (5'-GTG GTG CAG AAC TGC ATC TC-3') and bISG15-R (5'-GCC AGA ACT GGT CTG CTT GT-3'). GAPDH cDNA served as endogenous control, GAPDH was amplified using the primers bGAPDH-F (5'-AAC GGC ACA GTC AAG GCA GA-3') and bGAPDH-R (5'-TCG GCA GAA GGT GCA GAG AT-3'). The level of bISG15 transcription was normalized to GAPDH using the 2-ΔΔCt method. The polyclonal murine bISG15 antibody used in western blot assays was obtained in our lab. Western blot detection was performed using the chemiluminescence detection regent (Santa Cruz). To ensure equal protein loading, anti-β-actin antibody (Santa Cruz) was used to detect β-actin expression in cells.

**Figure 1:**
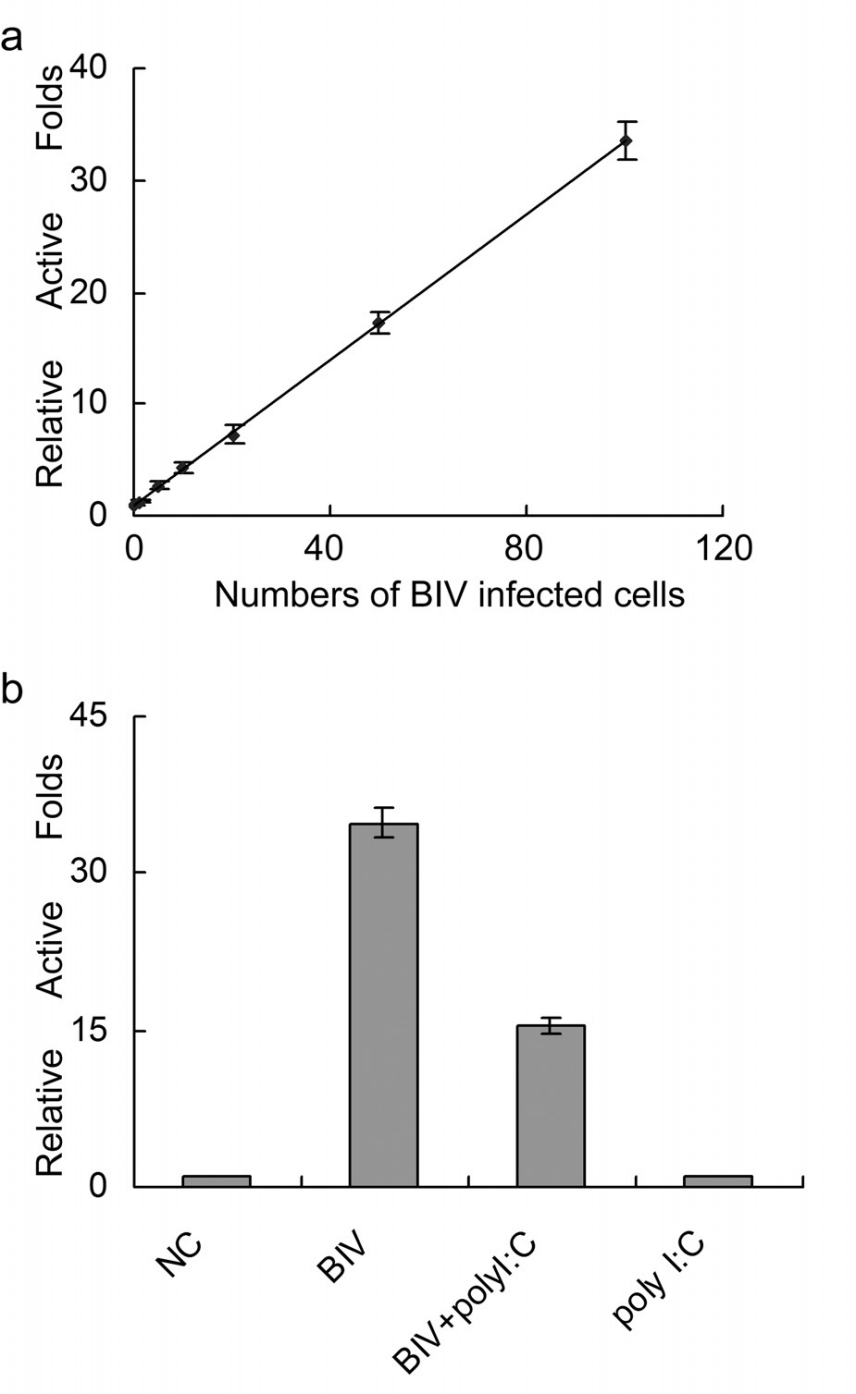
**The replication of BIV was repressed in poly I:C treated FBL cells**. a. BIV infection in FBL cells was measured quantitatively by BIV indicator cell line - BIVL cells. The × axis indicated the numbers of BIV infected cells; and the y axis indicated the relative luciferase activity folds. BIVL cells were plated at a density of 4 × 104 per well in 12-well plates. 1, 5, 10, 20, 50, 100 × 102 FBL cells were infected with BIV respectively, and normalized to the equal number (200 × 102) by adding naive FBL cells. The luciferase activity of adding only naive FBL cells was regarded as 1, and the relative luciferase activity folds of other groups were calculated. The relative folds were 1, 1.24, 2.73, 4.27, 7.23, 17.20, and 33.58. b. The replication of BIV in FBL cells indicated by relative luciferase activity in four experimental groups. The × axis indicates the four different experimental groups including the "NC" group (negative control), "BIV" group, "BIV + poly I:C" group, and "poly I:C" group; the y axis indicates the relative luciferase activity folds. The bars showed the relative luciferase activity folds of four groups respectively. All the experiments were carried out more than 3 times, the error bars are standard deviation (SD) from triplicate results.

In order to test the capacity indicative of BIV infection, different numbers of BIV-infected FBL cells were added to BIVL cells. To make the number of all FBL cells at equal level of 200 × 102, naive FBL cells free of BIV infection were added. Infected/naive FBL cells co-cultured with BIVL for 12 hours, and then the cells were taken luciferase assays. Naive FBL cells were added to the BIVL as negative control. As shown in Figure 1.a, the level of induced luciferase activity is in a dose dependent manner, which means the increased expressing level of luciferase activity is correlated with the increased numbers of BIV-infected FBL cells. It illustrated that BIVL reliably reveals BIV infection allowing infected cells to be tracked with high sensitivity. Thus, BIVL can act as an indicator cell line in this experiment. In the later experiments, the number of FBL cells infected BIV added in BIVL cells is about 100 × 102. In order to detect the effect of poly I:C treatment on BIV in FBL cells, the BIV infected FBL cells were divided into four groups as follows: "NC (negative control)" group, "BIV" group, "BIV + poly I:C" group and "poly I:C" group. Poly I:C was added in "BIV + poly I:C" group and "poly I:C" group, respectively, to reach a final concentration of 50 μg/ml at 2 hours before the BIV infection. BIV infection was performed in FBL cells in "BIV" group and "BIV+poly I:C" group, while mock-infection was performed in FBL cells in "NC" group and "poly I:C" group, respectively. After being infected for 12 hours, these cells were added to BIVL to perform luciferase activity assay. All the experiments were carried out more than 3 times. The luciferase activity of "NC" group was regarded as 1, and the relative luciferase activity folds of other groups were calculated divided by the activity of "NC" group. As shown in Figure 1.b, comparing with the "BIV" group, the replication of BIV was inhibited in FBL treated with poly I:C, whereas, no change of BIV replication was observed in the "NC" group nor did in the "poly I:C" group. It suggested that the treatment of poly I:C in FBL cells had no effect on the background activity of BIVL.

**Figure 2:**
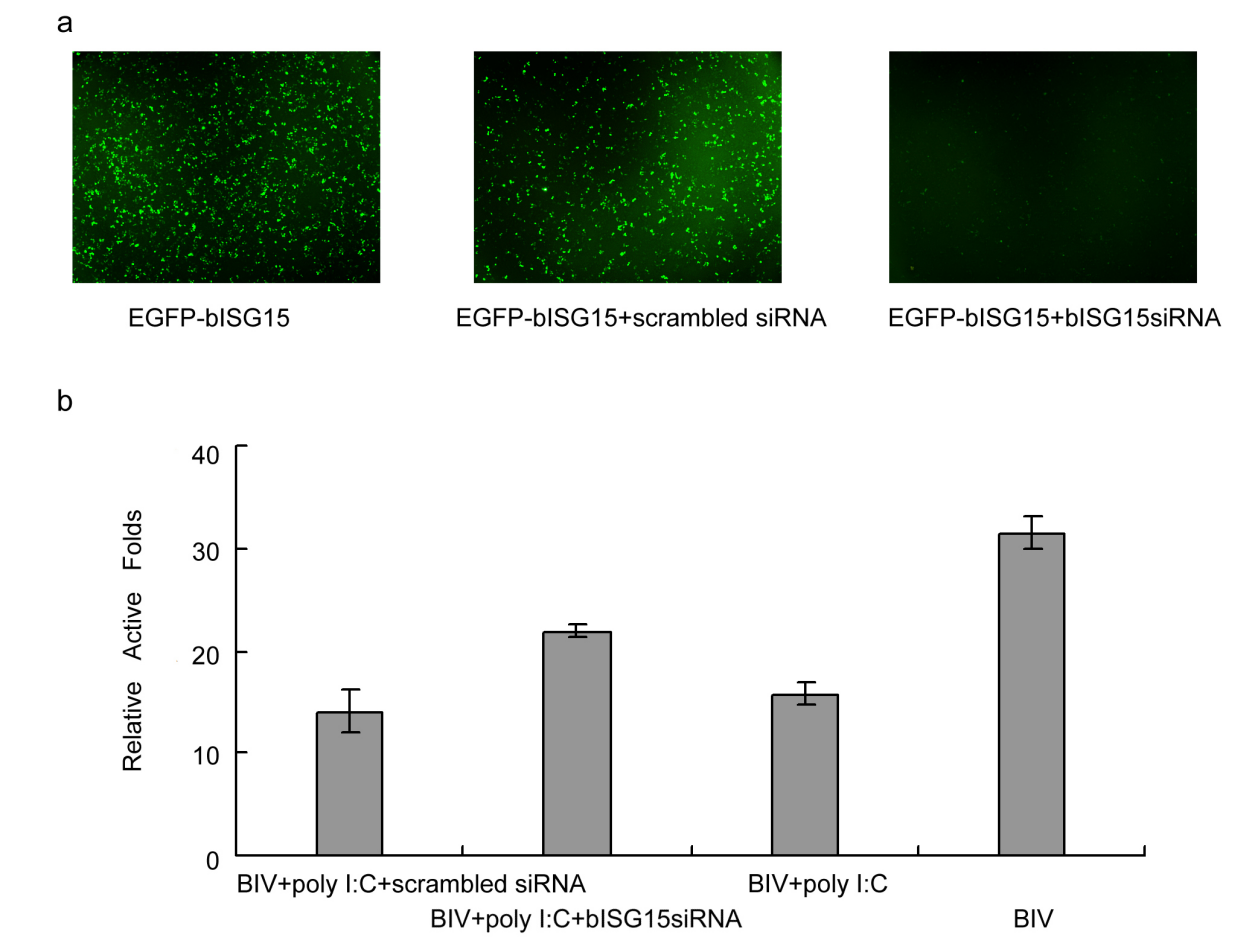
**bISG15 plays a role in poly I:C induced-inhibition of BIV in FBL cells**. a. The fluorescence microscopic image of 239T cells transfected with bISG15-EGFP expression plasmid, co-transfected with bISG15-EGFP expression plasmid and the scrambled siRNA, co-transfected with bISG15-EGFP expression plasmid and the bISG15siRNA respectively. b. bISG15siRNA can reverse the inhibition of BIV replication in FBL cells treated with poly I:C. The × axis indicates the four different experimental groups including the "BIV+ poly I:C+ scrambled siRNA" group, "BIV+ poly I:C + bISG15siRNA" group, "BIV + poly I:C" group and "BIV" group; the y axis indicates the relative luciferase activity folds. The bars show the relative luciferase activity of four groups, respectively. All the experiments were carried out more than 3 times, and the error bars are the standard deviation.

Poly I:C, which is the synthetic double-stranded RNA (dsRNA), can robustly induce synthesis of IFN or ISGs directly without the participation of IFNs [19]. TLR3, cytoplasmic RNA helicases RIG-1 and Mda-5, can recognize dsRNA and initiate the IRF-3 or IRF-7 signalling cascades, which sequentially induce transcriptions of some ISGs [20]. Poly I:C induces a number of ISGs with antiviral functions, and the antiviral ISGs contribute to the inhibition of BIV replication. To determine the critical role of bISG15 in poly I:C mediated inhibition of BIV, we designed the small interfering RNA (siRNA) of bISG15 gene. To demonstrate the siRNA can repress the expression bISG15 successfully, 239T cells were transfected with bISG15-EGFP expression plasmid only or co-transfected with bISG15-EGFP expression plasmid and the siRNA (scrambled siRNA or bISG15 siRNA). As shown in Figure 2.a, the siRNA of bISG15 can repress the expression of bISG15-EGFP specifically, and the scrambled siRNA can not effect the expression of bISG15-EGFP. In order to confirm our results, FBL cells infected with BIV served as a positive control, and untreated cells served as a negative control. FBL cells were transfected with bISG15siRNA or scrambled siRNA, followed by treatment with poly I:C. for 2 hours, then FBL cells were infected with BIV. After 12 hours post-infection, cells were added to BIVL to perform luciferase activity assay. The luciferase activity of negative control group was regarded as 1, relative luciferase activity folds of other groups were calculated as divided by the activity of the negative control group. As shown in Figure 2.b, these results suggested bISG15 siRNA reverse the inhibition of BIV in poly I:C treated FBL cells, whereas scrambled siRNA did not present such a role of reversion. Taken together, it suggested that bISG15 played a role in poly I:C-induced inhibition of BIV in FBL cells.

**Figure 3:**
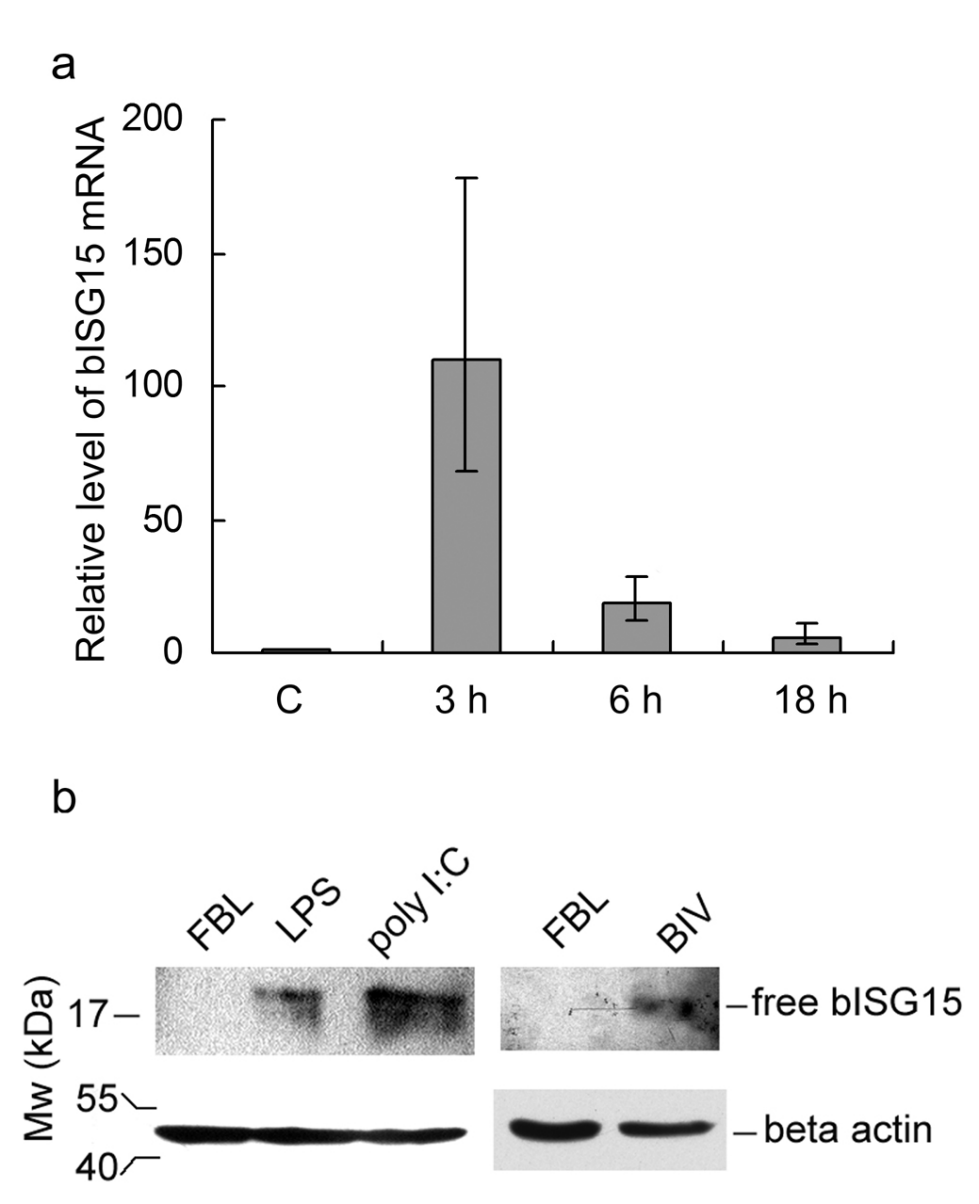
**The expression of bISG15 is induced in FBL cells infected with BIV**. a. The transcription of bISG15 increases in FBL cells infected with BIV. The × axis indicates experimental groups including FBL cells mock-infected, FBL cells infected with BIV at post-infection of 3 h, 6 h, and 18 h, respectively. The y axis indicates the relative folds of bISG15's mRNA level. b. bISG15 expression was detected in FBL cells treated with poly I:C or LPS and FBL cells infected BIV by western blot assay.

We demonstrated that bISG15 played a role in inhibiting the replication of BIV in FBL cells. In addition, we found the expression of bISG15 in naive FBL cells is very low, it is intriguing to address the question, and how the expression of bISG15 change in FBL cells after being infected with BIV. In order to validate whether the level of the bISG15 was increased in FBL cells with infection of BIV, bISG15 was detected by western blot using the antiserum against bISG15. FBL cells treated with poly I:C or LPS served as a positive control. FBL cells treated with PBS or mock-infected with naive FBL cells were used as negative controls. After 6 h, cells were collected to perform western blot. It was found that free bISG15 was detected in treated or BIV infected FBL cells, even though the expression level of bISG15 was not strong. In contrast, bISG15 positive bands were not observed in untreated or mock-infected FBL cells, as shown in Figure 3.b. We found that the level of bISG15 was increased in FBL cells treated with poly I:C/LPS or FBL cells infected with BIV. In order to detect the transcription of bISG15 during viral infection, total RNA from FBL cells infected with BIV were analyzed by real-time PCR. FBL cells infected with BIV were collected at 3 h, 6 h, and 18 h post infection and were carried out real-time PCR. As the negative control, FBL cells were mock-infected with an equal number of uninfected FBL cells. The bISG15's transcription was induced in FBL cells with infection of BIV as shown in Figure 3.a. These experiments confirmed that the expression of bISG15 is induced in FBL cells infected with BIV.

This study demonstrates that bISG15 is an antiviral and inducible protein in BIV infected fetal bovine lung cells. BIV infection can induce the expression of bISG15 in FBL cells, but it is still unclear that the detail involved in this process. It was shown in Figure 3.a, the induction of bISG15 in FBL cells infected with BIV was decreased at 3 hours post-infection, while FBL cells infected with BIV at 18 hours post-infection were still alive, not dead obviously. To infect the naive FBL cells, we added the BIV infected FBL cells to naive FBL cells. All cells involved in this experiment were exclusively FBL cells not other cells. FBL cells infected with BIV may activate some signal pathways like interferon, NF-κB signal pathway and so on. These signal pathways may induce the expression of bISG15 directly or some cytokines such as Type I interferon. These cytokines then induce the expression of bISG15. It indicates that the induction of bISG15's expression in FBL cells by BIV infection may be related to several viral and cellular events via unknown mechanism. Our results suggested that bISG15 played a role in poly I:C-induced inhibition of BIV in FBL cells. The function of bISG15 repressing the replication of BIV in FBL cells demonstrates that bISG15 is a key mediator induced in FBL cells inhibition of BIV replication and also reveals the antiviral function of ISG15 in other species.

## Competing interests

The authors declare that they have no competing interests.

## Authors' contributions

CL carried out all the experiments and drafted the manuscript. XL, XY, XK, WQ and YQ participated in the design of the study and revision of the manuscript. All authors read and approved the final manuscript.
